# What emotions are elicited by smells in Japanese people? Emotional measurement using a universal scale in Japanese

**DOI:** 10.1371/journal.pone.0323206

**Published:** 2025-05-13

**Authors:** Hirotada Hirama, Takahiro Miura, Shusuke Kanazawa, Yumeko Imamura, Shinya Kano, Tatsu Kobayakawa

**Affiliations:** 1 Human Augmentation Research Center, National Institute of Advanced Industrial Science and Technology, Kashiwa, Chiba, Japan; 2 Human Informatics and Interaction Research Institute, National Institute of Advanced Industrial Science and Technology, Tsukuba, Ibaraki, Japan; Universidad Autonoma de Chihuahua, MEXICO

## Abstract

Odors can elicit emotions, and cultural differences exist in the emotions elicited. To enable cross-cultural comparisons, the Universal Emotion and Odor Scales (EOS), a psychological scale consisting of affective terms in multiple languages, has been developed and used to measure emotions. However, it does not include the Japanese language. In addition, similar surveys only examined the pleasantness or unpleasantness of odors among Japanese people. No studies examined their relationship with specific emotions. We created a Japanese version of the EOS (the Japanese version of the Geneva Emotion and Odor Scale; J-GEOS), which could be compared across cultures. In addition, we conducted an experiment to examine the relationship between odors and emotions among Japanese participants. The J-GEOS was created by translating the existing multilingual EOS into Japanese. It was also used to examine emotional attraction to 10 odors in 200 participants, which included older adults. This study showed that the J-GEOS could be used to describe emotions elicited by odors via further specific affective terms. We expect that the J-GEOS could be widely used as a comparative tool between various cultures to understand the psychological characteristics of olfaction among the Japanese.

## 1. Introduction

Odors evoke emotions [1–9]. Emotions elicited by odors vary by ethnicity and culture [10,11]. Psychometric scales to measure emotions have been developed, such as the Emotion and Odor Scales (EOS), which uses affective terms for odors [12–14]. For cross-cultural comparisons, universal scales that can be applied to various nationalities and languages have been developed and used [15,16]. A representative EOS is the Universal Geneva Emotion and Odor Scale (UniGEOS) [14]. This scale allows for cross-cultural comparisons between Switzerland (French), the United States and the United Kingdom (English), Singapore and China (Chinese), and Brazil (Portuguese). However, there is no “universal scale” for the Japanese language. It is important to note that the Japanese and Chinese have quite different language systems in the following respects: characters, word reading, grammar, sentence structure (including word order), honorific and polite systems, and the number of words borrowed from multiple languages.

Studies have examined the relationship between odor and emotion in Japanese (Japanese speakers) from the perspectives of neuroscience [17] and perceptual psychology [18,19]. Research mainly focused on pleasant and unpleasant responses. Conversely, studies in Europe investigated the emotions elicited by odors and reported several specific emotions [14,20]. However, the relationship between various specific emotions and odors has not been reported in the Japanese population.

In this study, a Japanese version of the UniGEOS (the Japanese version of the Geneva Emotion and Odor Scale; J-GEOS) was created and used to measure emotions to allow for a cross-cultural comparison of the emotions elicited by odors. To conduct the experiment in a manner that emitted as little odor as possible into the surrounding environment, a mask-type olfactory presentation apparatus was used. This study was the first experimental investigation of the specific emotions elicited by odors among Japanese people.

## 2. Materials and methods

This study was conducted in accordance with the Declaration of Helsinki and its future amendments and was approved by the Ethics Committee of the National Institute of Advanced Industrial Science and Technology (Japan). All participants provided written, informed consent prior to inclusion.

### 2.1. Participants

Through preliminary screening, we selected 200 healthy Japanese participants who were born in Japan and were Japanese citizens. The inclusion criteria were those who had lived in Japan between the ages of 6 and 15 years, had not smoked in the past 12 months, and were aware of their proper olfactory function (Table 1). Exclusion criteria were set with respect to knowledge and body (Table 2). Years of living in Japan considered the fact that the emotions elicited by odors were influenced by cultural differences [14], which were differences in olfactory memory acquired from the environment. Specifically, we set the years of living in Japan based on the assumption that the participants were placed in a common olfactory environment during the period when mandatory schooling was required (aged 6–15 years in Japan). For screening, the Self-Administered Odor Questionnaire (SAOQ) [21] was administered to determine “awareness of proper olfactory function.” The SAOQ and olfactory Visual Analog Scale (VAS) used for the measurements are shown in S1 and S2 Appendices. The maximum score on the SAOQ was 40, but the full score varied depending on the participants’ responses. Scores relative to the full score were tabulated as percentages, of which scores above a certain level were considered eligible. In this study, those with a score of 70% or higher were considered eligible. The criterion (%) for this recruitment score was determined with reference to a previous study [21] of healthy participants with no history of nasal or sinus disease, in which participants who scored above 70% accounted for more than 90% of all participants. The VAS was answered by moving the cursor on the screen (S2 Appendix). In this study, those with a score of 70% or higher were considered eligible. Screening was conducted online on a separate date. Age categories of 65 years and older followed the literature [22]. To provide mental care to ineligible participants and avoid bias against eligible participants, participants were not informed of the results of the screening, reasons for passing or failing, and acceptance criteria. Participants were recruited in cooperation with AGEKKE CORPORATION (Japan) (the recruitment period: March 27, 2023 to April 4, 2023).

**Table 1:** Sample size, gender (% men), mean age (standard deviation (SD)), SAOQ score (SD), and olfactory VAS score (SD).

Age	Number (% men)	Mean age (SD)	% SAOQ score (SD)	% Olfactory VAS score (SD)
18-64 (“adults”)	100 (50)	51.0 (9.1)	100 (0)	88.6 (14.8)
65-74 (“pre-old age”)	80 (50)	69.5 (2.9)	100 (0)	86.9 (13.4)
≥ 75 (“old age”)	20 (50)	77.6 (2.5)	99.8 (0.8)	85.7 (14.0)

Participants were aged 29–83 years. SAOQ and VAS stand for Self-Administered Odor Questionnaire and Visual Analog Scale, respectively.

**Table 2:** Non-applicability requirements for screening.

Type	Non-applicability requirements
Knowledge-related	Those who have qualifications or licenses related to odors (olfactroy measurement operator, aroma, etc.)
	Those who belong to an odor-related organization (aroma environmental association, etc.)
Body-related (constitution and diseases)	Those who currently suffer from allergic rhinitis.
	Those who have been diagnosed with allergic rhinitis.
	Those who are currently undergoing treatment for allergic rhinitis.
	Those who currently suffer from sinusitis.
	Those who have been diagnosed with sinusitis
	Those who are currently undergoing treatment for sinusitis
	Those who have been diagnosed with olfactory disorders
	Those who have an allergy to smells.
	Those who have hypersensitivity to smells.
	Those who have difficulty making decisions due to dementia, etc.

### 2.2. Materials

#### 2.2.1. Japanese version of the Emotion and Odor Scale.

The affective terms in the UniGEOS were translated into Japanese to create a universal EOS. Among the four used languages, we translated the English and Chinese affective terms into Japanese. Japanese people generally had experience learning English, and Chinese was a language of a geographically close country and also an Asian language. Similar to UniGEOS, 25 emotion terms in nine categories were used in the translation. The translation was performed individually by two Japanese researchers via dictionaries and machine translation. Subsequently, each translation was compared to determine the most appropriate one. These procedures were partially based on a previous study [14]. However, the differences between UniGEOS and this study were as follows. First, in UniGEOS, the original term set was in English and was only subsequently translated from English into the target language, whereas in this study, it was translated from both English and Chinese. Second, in UniGEOS, a coordinator experimenter who was not involved in the translation was involved in checking the correspondence between the original and translated term sets, whereas in this study, the experimenter (not the translator) who checked the correspondence did not participate. This is because the set of terms translated in this study (25 terms) is much smaller than that in UniGEOS (about 480 terms), which we thought could be simplified. Nonetheless, the following aspects of the translation procedure used in this study were the same as those used in UniGEOS. First, both were translated by multiple people. Second, both were translated from the original language to the target language and then translated back into the original language. Table 3 shows the Japanese version of the EOS translated based on the UniGEOS. We named the list of affective terms “J-GEOS.”

**Table 3:** The UniGEOS and Japanese translations of the affective terms (J-GEOS).

Affective categories		Affective terms				
		English	French	Chinese	Portuguese	Japanese
A.	Unpleasant feelings	Disgusted	DégoÛté	厌恶的	Enojado	**むかむかする**
		Irritated	Irrité	恼怒的	Irritado	**イライラする**
		1	Désagréablement surpris	不愉快的意外惊喜	Desagradavelmente surpreso	**ぎょっとする（不快な驚き）**
B.	Happiness/Delight	Happy	Heureux	幸福的	Feliz	**幸せな**
		Pleasantly surprised	Agréablement surpris	惊喜的	Agradavelmente surpreso	**驚き喜ぶような**
		Well-being	Bien-être	安宁	Bem-estar	**心が落ち着く**
C.	Sensuality/Desire	Desire	Désir	渴望	Desejo	**心がひかれる**
		Romantic	Romantique	浪漫的	Romântico	**ロマンチックな**
		Sensual	Sensuel	肉欲的	Sensual	**官能的な**
D.	Energy	Refreshed	Rafraîchi	恢复精神的	Refrescado	**爽快な**
		Energetic	Energique	精力充沛的	Energético	**エネルギッシュな**
		Revitalized	Revitalisé	恢复生机的	Revitalizado	**元気が回復する**
E.	Soothing/Peacefulness	Relaxed	Relaxé	得到安宁的	Relaxado	**ゆったりした**
		Comforted	Réconforté	宽慰的	Confortado	**心地よい**
		Soothed	Apaisé	受安慰的	Sossegado	**癒される**
F.	Hunger/ Thirst	Mouth-watering	Salivant	令人垂涎欲滴的	Com água na boca	**食欲をそそる**
		Thirsty	Assoiffé	口渴的	Sedento	**喉が渇くような**
		Famished	Affamé	极饥饿的	Faminto	**空腹を感じる**
G.	Interest	Amusement	Amusement	娱乐	Diversão	**楽しい**
		Interesting	Captivant	有趣的	Interessante	**面白い**
		Impressed	Impressionné	印象深刻的	Impressionado	**感激するような**
H.	Nostalgia	Sad	Triste	伤心的	Triste	**悲しい**
		Melancholic	Mélancolique	忧郁的	Melancólico	**憂うつな**
		Nostalgic	Nostalgique	怀旧的	Nostálgico	**懐かしい**
I.	Spirituality	Spiritual feeling	Sentiment spirituel	精神感觉	Sentimento espiritual	**スピリチュアルな**

Affective categories and terms in English, French, Chinese, and Portuguese are reproduced from UniGEOS [14]. The consecutive letters A to I beside the affective categories are assigned by the authors for convenience.

#### 2.2.2. Olfactory presentation apparatus.

**Fig 1:**
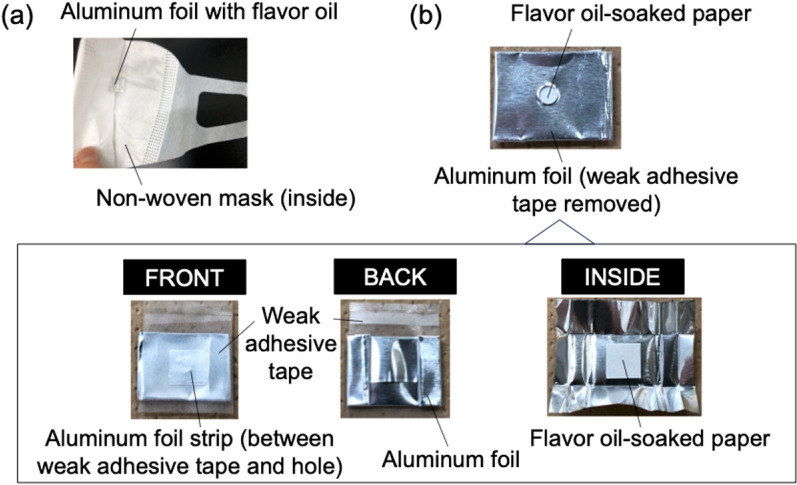
Fabricated mask-type olfactory presentation apparatus. (a) Non-woven mask with aluminum foil with flavored oil fixed inside; (b) aluminum foil with flavored oil. In the figure above, the weak adhesive tape is removed as in use (when sniffing). When fixing it to the non-woven mask, it is first folded in half lengthwise and then fixed. In the figure below, the interior and exterior views are shown.

A mask-type olfactory presentation apparatus was fabricated (Fig 1). One fragrance was used (applied) per mask and placed inside the nonwoven mask. To ensure that the odor did not leak until the mask was used, various steps were performed. A piece of paper was soaked with a drop of fragrance (20 µL) and covered with aluminum foil (a material with almost no odor permeability) with a 1 mm hole. A weak adhesive tape was applied to cover the hole (removed when the mask was used). Double-sided adhesive tape (a material with almost no odor) was applied to the side of the aluminum foil opposite the hole. Furthermore, this was fixed to the non-woven mask. The masks were placed individually in an odor-resistant plastic bag, and 10 masks with different odors were placed in a large plastic bag (for an experiment in which each person smelled 10 different odors). To facilitate identification during data aggregation, each mask was marked with a symbol that corresponded to the odor type. A preliminary study on the retention of odor intensity showed that the masks retained their odor intensity for at least three months. In addition, there was almost no leakage outside the bag.

#### 2.2.3. Odors.

In total, 10 commercial fragrances were used (Table 4). The fragrances were selected from previous studies on odors and emotions [10] and reference odors commonly used in odor judgments according to the International Standard Organization (ISO) 12219–7:2017 [23]. Similar to a previous study [14], the fragrances were selected from both pleasant and unpleasant and food-related and non-food-related odors. They were diluted with an odorless mineral oil. Based on a preliminary study, the concentration and amount of fragrance were set to achieve an odor intensity within human perception. Specifically, the odor was set between the range of “weak odor that could be recognized as what it was” and “odor that could be easily perceived” in the six-step odor intensity display method (under the jurisdiction of the Ministry of the Environment in Japan). The odors were presented in a random order.

**Table 4:** Odor characteristics used in this study.

Identifier	Flavor compound	Organoleptic	Concentration (%)
1	γ-Undecalactone	Peach	1
2	Skatole	Feces	1 x 10^–2^
3	2,4-Decadienal	Meaty, citrus	3
4	Undecanal	Fatty, rose	1
5	Ethyl isobutyrate	Ethereal, fruity	1 x 10^1^
6	Benzaldehyde	Almond	1
7	δ-Decalactone	Milk	1
8	Geosmin	Distinct earthy, musty	1
9	Cinnamaldehyde	Cinnamon	1
10	Citronellol	Citrus, floral	3

Concentrations are shown by mass for skatole only and volume for all others. γ-Undecalactone, 2,4-decadienal, ethyl isobutyrate, benzaldehyde, geosmin, citronellol were purchased from FUJIFILM Wako Pure Chemical Corporation (Japan). Skatole and cinnamaldehyde were purchased from Sigma-Aldrich (USA). Undecanal was purchased from Tokyo Chemical Industry Co., Ltd. (Japan). δ-Decalactone was purchased from Thermo Fisher Scientific (USA).

### 2.3. Procedure

Screened participants performed the experiment using various olfactory presentation apparatuses. An olfactory presentation apparatus in the bag was removed, worn, and sniffed for five seconds (no information on the odor was provided to the participant). Subsequently, the apparatus was removed and placed back in the bag, which was closed to prevent odor leakage. Participants were allowed three minutes to answer the J-GEOS terms shown in Table 3 (if they completed the scales within three minutes, they were provided a break until three minutes elapsed to avoid odor fatigue). Participants rated 25 affective terms on a 5-point Likert scale that ranged from “not at all” to “very strongly.” This process was repeated for all fragrance types. Each experimental session lasted for one hour. In total, 10 paper-based question sets with different sequences of affective terms were prepared. These were bundled in random order and distributed to each participant as a set. They were asked to refrain from consuming food or drinks (not water) one hour prior. This experiment was conducted in cooperation with AGEKKE CORPORATION and in April 2023 in Tokyo, Japan, in a typical conference room.

### 2.4. Analysis

We employed exploratory and confirmatory factor analyses to answer the question items. First, we tried to remove items for which reliability could not be assured using the Kaiser-Meyer-Olkin measure of sampling adequacy. Subsequently, we employed Bartlett’s sphericity test to confirm the lack of correlation among the observed variables. Consequently, all indices were sufficiently valid (Cronbach’s α > 0.9 and *p* < 0.05), which indicated that reliability was confirmed. An appropriate number of factors were verified via the Kaiser-Guttman criterion, scree plot, parallel analysis, MAP (minimum average partial) test, and parallel analysis with squared multiple correlation (SMC). The factors were extracted via the maximum likelihood method, and the factor axes were rotated via promax rotation. Finally, the number of factors extracted via the exploratory factor analysis was determined by subjective interpretability and goodness-of-fit measures, such as the root mean square error of approximation (RMSEA) and Tucker-Lewis index (TLI). Furthermore, we examined the conditions with items with factor loadings of 0.4 or more for each factor and when factor loadings were reasonable to interpret, with reference to the results reported by Ferdenzi et al. [14]. This cutoff value of 0.4 for the factor loadings was determined based on the sample size, as adopted and recommended by various literature [24–27]. Next, a confirmatory factor analysis was performed to confirm the validity of the extracted factors. In this process, we used goodness of fit indices such as RMSEA, TLI, comparative goodness of fit index (CFI), and standardized root mean square residual (SRMR).

We calculated factor scores using a regression approach based on the factor loadings obtained from the factor analysis [28]. Each factor’s mean score was 0, with a standard deviation 1 [29]. The obtained factor scores were then grouped by odor type and analyzed for significance relative to the mean (equal to 0) using a one-sample t-test. This analysis will explain how each odor is characterized concerning the factor scores.

## 3. Results

### 3.1. Latent factor extraction

Table 5 (and S1 Table) depict the factor loading matrix and factor correlation matrix of the J-GEOS obtained by factor analysis, respectively. We conducted a factor analysis for the 3–9 factors of the factor numbers criteria. We extracted seven factors based on interpretability: (1) happiness/soothing/attraction, (2) unpleasant/anxiety, (3) vitality, (4) hunger/thirst, (5) sensuality/desire, (6) nostalgia, and (7) spirituality (RMSEA = .034 < .05, TLI = .985 > .95). In the confirmatory factor analysis, RMSEA = .059 < .10, TLI = .985 > .95, CFI = .988 > .95, and SRMR = .060 < .10.

**Table 5:** Factor loading matrix of the J-GEOS via exploratory factor analysis.

Affective category		Affective term (English)	Extracted factor						
			ML1	ML2	ML3	ML4	ML5	ML6	ML7
A.	Unpleasant feelings	Disgusted		0.92					
		Irritated		0.95					
		Unpleasantly surprised		0.81					
B.	Happiness/Delight	Happy	0.89						
		Pleasantly surprised			0.40				
		Well-being	0.90						
C.	Sensuality/Desire	Desire	0.75						
		Romantic	0.60				0.63		
		Sensual					0.72		
D.	Energy	Refreshed	0.72						
		Energetic			0.71				
		Revitalized	0.70						
E.	Soothing/Peacefulness	Relaxed	0.76					0.41	
		Comforted	1.01						
		Soothed	0.93						
F.	Hunger/Thirst	Mouth-watering				0.70			
		Thirsty				0.45			
		Famished				0.98			
G.	Interest	Amusement	0.69						
		Interesting			0.44				
		Impressed							
H.	Nostalgia	Sad		0.58					
		Melancholic		0.80					
		Nostalgic						0.58	
I.	Spirituality	Spiritual feeling							0.43
**Interpreted factors**			**Happiness/Soothing/Attraction**	**Unpleasant/Anxiety**	**Vitality**	**Hunger/Thirst**	**Sensuality/Desire**	**Nostalgia**	**Spirituality**
Cronbach’s α			0.97	0.903	0.78	0.82	0.84	0.93	0.72
Proportional contribution ratio			0.32	0.14	0.08	0.07	0.06	0.04	0.02
Cumulative contribution ratio			0.32	0.46	0.53	0.61	0.67	0.70	0.72

Affective terms are in Japanese; however, they are displayed in English for convenience.

The categories to which the affective terms belong were considered to assign names to each factor. Particularly, affective terms that are highly related to Factor 1 were all in the category “E. Soothing/Peacefulness”; “Happy” and “Well-being” belonged to “B. Happiness/Delight”; “Desire” and “Romantic” belonged to “C. Sensuality/Desire”; “Refreshed” and “Revitalized” belonged to “D. Energy”; and “Amusement” belonged to “G. Interest.” Thus, this factor was named “happiness/soothing/attraction” to account for the “soothing/peacefulness” and “happiness/delight” categories. The last word in the name, “attraction,” integrates the terms “desire,” “romantic,” “refreshed,” “revitalized,” and “amusement.” Factor 2 shows high loadings for all affective terms in “A. Unpleasant feelings,” while “melancholic” and “sad” belonged to “H. Nostalgia.” Thus, this factor was named “Unpleasant/Anxiety.” The word “anxiety” integrates the “melancholic” and “sad” feelings.

Factor 3 was composed of the term “energetic,” which belonged to “D. Energy”; “interesting” belonged to “G. Interest”; and “pleasantly surprised” belonged to “B. Happiness/Delight.” The factors could be independent of the existing affective category and was named as “Vitality” because the constitutive emotion was mainly “energetic” and related to an active nature whose elements were “interest” and “pleasant surprise.” Affective terms related to Factor 4 belonged to the “F. Hunger/Thirst” category. Therefore, this factor was directly termed the “Hunger/Thirst” factor.

Regarding Factor 5, the factor loadings for Desire were indeed less than 0.4, and the factor loadings did not completely match the Sensuality/Desire category. However, because Ferdenzi et al. [14] used the category name as the factor name even in such a case, we decided to follow their precedent and name the factor Sensuality/Desire. Factor 6 was comprised of “nostalgic” belonging to “H. Nostalgia,” and “Relaxed” belonging to “E. Soothing/Peacefulness.” Thus, we named this factor as “nostalgia,” more so because several studies have reported its co-occurrence with relaxation in nostalgia [30,31]. The last factor, Factor 7, we named ‘spirituality” because it related solely to “spiritual feeling,” which belonged to “I. Spirituality.”

The results, summarized in Table 5, indicated that the extracted factors were partially consistent with the UniGEOS subscales. In particular, the factor loadings of ML2, ML4, ML5, and ML7 were high for multiple affective terms in a single affective category. Meanwhile, the factor loadings for ML1, ML3, and ML6 showed high factor loadings across multiple affective categories. However, similar to the study by Ferdenzi et al. [14], since the affective categories with high factor loadings differed by country and internal validity parameters were within the acceptable range, our results could reasonably ascertain the validity of the olfactory components.

### 3.2. Cross-cultural comparison of factors

Factors were extracted and compared with those of other cultures (Table 6 and S2 Table (colored version of Table 6)). The extracted factors for each region were obtained from the results of the UniGEOS study [14]. Generally, the results indicated that the extracted factors shared some similarities with those observed in other cultures. Particularly, factors related to “Disgust/Irritation” (or “Unpleasant/Anxiety,” colored red in S2 Table) and “Sensuality/Desire” (purple) appeared to have some degree of resemblance or association with those found in other cultures, despite some differences. Moreover, factors resembling or associated with “Happiness/Well-being” (pale green) were extracted as “Happiness/Soothing/Attraction” (pale green and forest green). A “Vitality” factor (pink) similar or related to the “Energy” factor (pink) was also identified.

**Table 6:** Cross-cultural comparison of extracted factors.

	Europe		North and South America			Asia		
	Geneva, CH	Liverpool, UK	Fayetteville, AR, USA	Davis, CA, USA	Campinas, BR	Beijing, CN	Singapore, SG	Tokyo, JP
1	Disgust/Irritation	Disgust/Irritation	Disgust/Irritation	Disgust/Irritation	Disgust/Irritation	Disgust/Irritation	Disgust/Irritation	**Happiness/Sooth-ing/Attraction**
2	Happiness/Well-being	Happiness/Well-being	Happiness/Well-being	Happiness/Well-being	Happiness/Well-being	Happiness/Well-being	Happiness/Well-being	**Unpleasant/Anxiety**
3	Sensuality/Desire	Sensuality/Desire	Sensuality/Desire	Sensuality/Desire	Sensuality/Desire	Sensuality/Desire	Sensuality/Desire	**Vitality**
4	Energy	Energy	Energy	Energy	Energy	Energy	Energy	**Hunger/Thirst**
5	Soothing/Peacefulness	Soothing/Peacefulness	Soothing/Peacefulness	Soothing/Peacefulness	Soothing/Peacefulness	Soothing/Peacefulness	Negative feelings	**Sensuality/Desire**
6	Sensory pleasure	Hunger/Thirst	Hunger/Thirst	Hunger/Thirst	Hunger/Thirst	Arousal	Intellectual stimulation	**Nostalgia**
7		Nostalgia		Negative feelings	Nostalgia	Melancholy	Sprituality	**Sprituality**

Reprinted from a previous study on the UniGEOS [14], except for Japan. In S2 Table, factors with similar or related characteristics are presented in the same color font.

Unlike other East Asian countries, such as Beijing (China) and Singapore, “Hunger/Thirst” (blue) was extracted in Tokyo. Furthermore, we observed that “Nostalgia” (orange) was extracted and identified only in the UK and Brazil. Beijing also had a somewhat similar factor to Tokyo. However, the seventh factor, “Melancholy” (orange), consisted of loneliness and nostalgia. Conversely, “Nostalgia” included nostalgia and relaxed feelings. “Spirituality” (grey), identified only in Singapore, was also extracted from our study. These commonalities between Japan and other cultures may suggest that Japanese participants perceived odor-related factors in ways that are somewhat similar to those in other cultures.

### 3.3. Relationship between odor and the factor score

**Fig 2:**
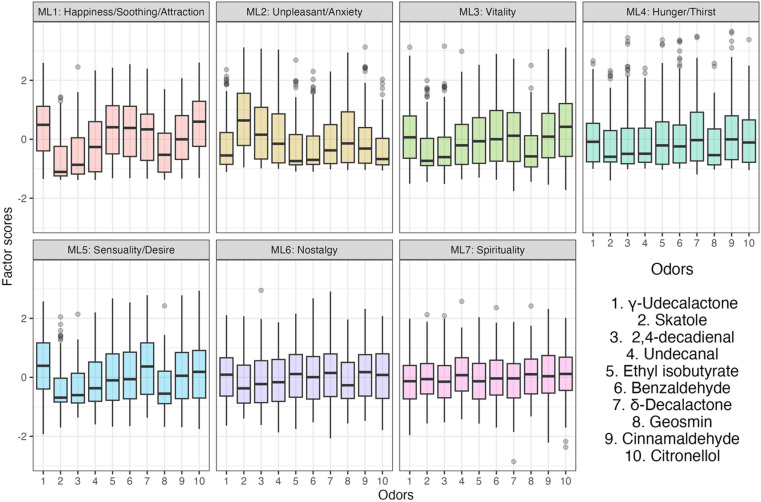
Factor scores for the odors in each extracted factor.

**Fig 3:**
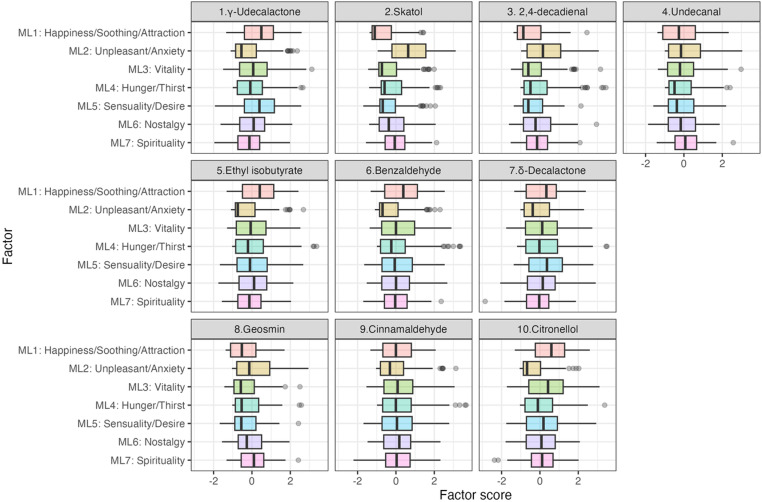
Factor scores for the extracted factors in each odor.

Figs 2 and 3 show the factor scores of the odors for each extracted factor and the extracted factors for each presented odor, respectively. We found that the scores for the factors with higher contributions could generally show greater variability. Table 7 shows whether the scores are significantly higher or lower than the factor score. The combinations of significantly higher and lower factor scores were different for all the presented odors. Particularly in the factor scores of ML1 (happiness/soothing/attraction) and ML2 (unpleasant/anxiety), which generally had high contribution rates, the former was significantly higher and the latter significantly lower for the favorable odors, such as 1, 5, 6, 7, and 10. Meanwhile, the opposite trend was observed for the unfavorable odors, such as 2, 3, 4, and 8. The factor scores of factors with low contribution rates, such as ML6 (nostalgia) and ML7 (spirituality), were significantly lower in the presented odors. Therefore, the extracted factors might be able to characterize the subjective impression that Japanese people perceived for each odor. Furthermore, the results obtained may enable to confirm the construct as well as its internal validity.

**Table 7:** Comparison of the factor scores for odors in each extracted factor.

ID	Odor	Higher or lower factor scores as compared to 0						
		ML1	ML2	ML3	ML4	ML5	ML6	ML7
		Happiness/Soothing/Attraction	Unpleasant/Anxiety	Vitality	Hunger/Thirst	Sensuality/Desire	Nostalgia	Spirituality
1	γ-undecalactone	**H**	L		**H**	**H**	L	
2	scatole	L	**H**	L	L	L		L
3	2,4-decadienal	L	**H**		L	L	(L)	L
4	undecanal	L	**(H)**	L	L		**(H)**	
5	ethyl isobutyrate	**H**	L				(L)	
6	benzaldehyde	**H**	L			**H**		
7	delta-decalactone	**H**		**H**	**H**	**H**		**(H)**
8	geosmin	L	**H**	L	L	L	**(H)**	(L)
9	cinnamaldehyde			**H**	**H**	**H**		**(H)**
10	citronellol	**H**	L		**H**	**H**	**H**	

**H**: Signifincatly higher than 0 (p < 0.05), **(H)**: Higher than 0 with marginal significance (p < 0.10). L: Signifincatly lower than 0 (p < 0.05), (L): Lower than 0 with marginal significance (p < 0.10). [Blank]: Nonsignificant compared to 0 (p ≥ 0.10). The magnitude and significance of the factor scores were evaluated by comparing them to 0 via a one-sample t-test.

## 4. Discussion

### 4.1. The J-GEOS and its limitations

This study added the Japanese language to the EOS, which included the UniGEOS. This could have allowed us to compare Japanese people (Japanese speakers) with those of several other representative cultures. In addition, Japanese people may be able to express pleasant and unpleasant feelings and also specific self-reported feelings regarding the emotions elicited by odors.

There are two main limitations of the J-GEOS: the EOS in general and the J-GEOS in particular. According to Ferdenzi et al. [14], who created the UniGEOS, the EOS (which included the UniGEOS) had the following limitations. First, the affective terms in the EOS did not capture all of the emotion components (which included behavioral tendencies, physiological arousal, cognitive processes, and expressive motor behavior) [32]. To examine true emotions, the scale must be fully validated by adding cognitive, behavioral, and physiological perspectives [33]. Second, the developed scale did not measure a specific group of products. Products had many different sensory and economic aspects [34–36]. Hence, it was impossible to measure emotional responses as a whole. Third, odor-related memories could cause uncontrolled variations in emotional responses. Finally, only specific populations (or cultures) were included. Inter-relationships with other populations, such as Spanish-speaking or African cultures, could not be observed. Conversely, the J-GEOS-specific limitations included difficulty in fully adapting Japanese terminology to UniGEOS, which began as a European language. “Well-being” was a sense that did not exist in the Japanese language and was newly imported recently. Thus, it is necessary to consider the difficulty of comparing sensory and affective terms that are unfamiliar to Japanese people. In addition, since the participants were generally sensitive to odors, it was unclear which characteristics would emerge with a bias toward feelings. It should also be noted that it is not possible to make a simple comparison between these because of the methodological differences between the current study and that by Ferdenzi et al. [14].

### 4.2. Similarities and differences between the cultures

Some differences between the current and previous studies using UniGEOS [14] should be considered. In this study, the participants sniffed 10 predetermined odor samples. Conversely, the previous study was an experimental system in which participants selected and sniffed odor samples from a prepared list. Specifically, in the preliminary experiment for the construction of the UniGEOS, 14 odor samples were selected from 24 odor samples. Furthermore, in the validation experiment that used the constructed UniGEOS, 7–8 odor samples were selected from 56–59 odor samples. Considering the different fragrances used, there is a possibility that the different fragrances may have affected the results. As with the discussion in Section 4.1, a simple comparison of the discussion in this section with Ferdenzi et al. [14] is not possible due to methodological differences.

Common, similar and related factors could be found in Japanese and other cultures (Table 6). These findings might partially support the idea that affective responses elicited by odors share some degree of commonality across cultures, as noted in previous studies [11,13,14,37–40]. Meanwhile, all these prior studies have pointed out that the classification of odors and the emotions elicited by them can be divided into core representations common to different cultures, and more culturally specific representations. For the core representations, some studies have reported aspects of pleasant and unpleasant affect excitation related to odor perception [37,39,41]. Those findings align to some extent with our results, especially Disgust/Irritation (or Unpleasant/Anxiety in this study) and Happiness/Well-being (or Happiness/Soothing/Attraction in this study) as well as Sensuality/Desire, which can be considered close to pleasantness and unpleasantness. These responses seem to be common to various cultures, including Japan. Similarly, the elicitation of Hunger/Thirst responses by odors has also been reported as a phenomenon observed across multiple cultures [14,42,43], a point which is also reflected in our results. Stevenson proposed that people share three common olfactory functions (related to risk avoidance, feeding, and social communication) [44]. It can be plausible that the factors related to these three functions emerged in both the Japanese and other cultural contexts. However, it should be noted that although similar factors were elicited across different cultures, the specific makeup of these factors differed. Thus, further study is required to clarify these differences in detail.

We discuss the cultural similarities at the four points above in more detail. Regarding Disgust/Irritation (or Unpleasant/Anxiety in this study), it has been reported that aversive reactions to specific odors, particularly body odor, are relatively universal across cultures. Body odor is thought to evoke strong feelings of disgust, regardless of culture [45]. This suggests that there is a common biological basis for aversive responses to odors. The Body Odor Disgust Scale was developed to assess individual differences in sensitivity to disgust of body odor [46]. The validity of the scale in multiple studies indicates that there is a certain degree of consistency in people’s reactions to unpleasant body odors. In particular, unpleasant odors associated with illness seem to cause similar physiological and immunological reactions in all individuals [47]. This suggests an evolutionarily conserved mechanism for sensing and avoiding the threat of pathogens through a sense of smell. Regarding happiness/well-being (or happiness/soothing/attraction in this study), the effects of pleasant scents on mood improvement and anxiety reduction have been investigated in different cultures. Specifically, pleasant smells have been shown to improve positive emotions and comfort/happiness [48,49], reduce anxiety levels [48,49], and improve overall mood [50,51]. These effects have been observed to be largely consistent across cultures. In addition, it has been reported that deeper familiarity with a scent is accompanied by more positive emotional responses, regardless of culture [52]. The effects of pleasant scents on mood enhancement are thought to occur through several mechanisms. (1) Autobiographical memory: Scents that evoke personally pleasant memories are particularly effective in enhancing mood [49]. (2) Physiological changes: Pleasant smells can trigger a relaxation response such as a decrease in heart rate and an increase in skin conductivity [49]. (3) Effects on the nervous system: Smelling pleasant smells activates areas of the brain associated with positive emotions and memories [48]. (4) Learning associations: Regular exposure to pleasant scents in the natural environment may reduce stress response over time [51]. Regarding Sensuality/Desire, multiple studies have shown that the relationship between olfaction and sexual desire is consistent across cultures. Cross-cultural studies conducted in China, India, and the United States have found that regardless of cultural background or gender, people who value smells and like to smell other people’s body odors have stronger sexual desires [53]. Sense of smell influences sexual desire and behavior by detecting pheromones and other chemical signals [54]. Regarding Hunger/Thirst, regardless of food culture, the smell of food stimulates appetite and influences eating behavior. The ability of smells to evoke hunger appears to be based on a learned association between smell and the consequences of food intake. Humans can extract nutritional information from food cues through olfaction, which triggers specific appetites and promotes food selections [55]. Some of the neurobiological mechanisms of hunger induced by smell have been identified. A signaling molecule called neuropeptide Y (NPY) plays an important role in promoting attraction to the smell of food over other olfactory cues [56]. NPY is secreted by neurons that control hunger in the thalamus, which controls the processing of sensory information.

Regarding the cross-cultural differences in affective responses, the factors of nostalgia and spirituality, which have been identified in specific cultures, were also found in the present study. Concerning the nostalgia factor, Gotow et al. [57] suggested that nostalgia could arise in Japanese food culture due to the focus on seasonal variations. Moreover, some studies have reported on the use and effectiveness of reminiscence therapy, mainly utilizing food odors, for older adults in Japan [58,59]. This cultural context may have influenced our findings, especially as more than half of the participants in this study were older adults. This last fact, the preponderance of older adults in this study, may have also affected the emergence of the spirituality factor. For instance, it has been shown that older adults tend to manifest a more positive spirituality [60]. It is also possible that the fragrance used in this study may have influenced the extraction of the spirituality factor. For instance, only cinnamaldehyde showed a tendency to elicit “spirituality” (found only in Singapore) among the fragrances used in this study. This compound was responsible for the odor of cinnamon, which was sometimes found in Buddhist incense. In Japan and Singapore, approximately 50% and 30% of the population are Buddhists, respectively. Furthermore, in China and other parts of Eastern Asia, Buddhists comprised approximately 20% of the population (2022 Report on International Religious Freedom, published by the US government). These findings suggested that “spirituality” might emerge as a factor in cultures with relatively large Buddhist populations, Buddhist populations, which was consistent with a previous study [14]. Although sensitivity to odor could differ among different ethnic groups [61], there may be some commonalities among ethnic groups that are relatively geographically close to each other. Further research is required to determine the possibility of the appearance of such factors, and how they should be weighed.

## 5. Conclusion

This study created a Japanese version of the EOS (J-GEOS) to measure emotions and compare the Japanese and other cultures regarding the elicitation of emotions by odors. In terms of the use of mask-type olfactory presentation apparatus, this study demonstrated its potential applicability in the measurement of emotions. However, further basic and applied knowledge related to this apparatus should be accumulated for the development of personalized olfactory presentations. In addition, a more detailed analysis of the present study may help to uncover attribute-specific characteristics of Japanese individuals. The J-GEOS may prove useful as a comparative tool for examining olfactory psychological traits across various cultures, particularly in relation to understanding the Japanese people. Moreover, from the perspective of odor-related product development, the findings of this study could potentially inform the creation of new products that are more suitable for Japanese people as well as for people with a similar cultural background.

## Supporting information

S1 Appendix Self-administered odor questionnaire. (PDF)

S2 Appendix Olfactory Visual Analog Scale. (PDF)

S1 Table Factor-correlation matrix. (PDF)

S2 Table Cross-cultural comparison of extracted factors (colored version of Table 6). (PDF)
