# Correction: What emotions are elicited by smells in Japanese people? Emotional measurement using a universal scale in Japanese

**DOI:** 10.1371/journal.pone.0340419

**Published:** 2026-01-05

**Authors:** The PLOS One Staff

There are errors in Table 3.

The publisher apologizes for the error.

**Table 3:** The UniGEOS and Japanese translations of the affective terms (J-GEOS).

Affective categories		Affective terms				
		English	French	Chinese	Portuguese	Japanese
A.	Unpleasant feelings	Disgusted	Dégoûté	厌恶的	Enojado	**むかむかする**
		Irritated	Irrité	恼怒的	Irritado	**イライラする**
		Unpleasantly surprised	Désagréablement surpris	不愉快的意外惊喜	Desagradavelmente surpreso	**ぎょっとする** **(不快な驚き)**
B.	Happiness/ Delight	Happy	Heureux	幸福的	Feliz	**幸せな**
		Pleasantly surprised	Agréablement surpris	惊喜的	Agradavelmente surpreso	**驚き喜ぶような**
		Well-being	Bien-être	安宁	Bem-estar	**心が落ち着く**
C.	Sensuality/ Desire	Desire	Désir	渴望	Desejo	**心がひかれる**
		Romantic	Romantique	浪漫的	Romântico	**ロマンチックな**
		Sensual	Sensuel	肉欲的	Sensual	**官能的な**
D.	Energy	Refreshed	Rafraîchi	恢复精神的	Refrescado	**爽快な**
		Energetic	Energique	精力充沛的	Energético	**エネルギッシュな**
		Revitalized	Revitalisé	恢复生机的	Revitalizado	**元気が回復する**
E.	Soothing/ Peacefulness	Relaxed	Relaxé	得到安宁的	Relaxado	**ゆったりした**
		Comforted	Réconforté	宽慰的	Confortado	**心地よい**
		Soothed	Apaisé	受安慰的	Sossegado	**癒される**
F.	Hunger/ Thirst	Mouth-watering	Salivant	令人垂涎欲滴的	Com água na boca	**食欲をそそる**
		Thirsty	Assoiffé	口渴的	Sedento	**喉が渇くような**
		Famished	Affamé	极饥饿的	Faminto	**空腹を感じる**
G.	Interest	Amusement	Amusement	娱乐	Diversão	**楽しい**
		Interesting	Captivant	有趣的	Interessante	**面白い**
		Impressed	Impressionné	印象深刻的	Impressionado	**感激するような**
H.	Nostalgia	Sad	Triste	伤心的	Triste	**悲しい**
		Melancholic	Mélancolique	忧郁的	Melancólico	**憂うつな**
		Nostalgic	Nostalgique	怀旧的	Nostálgico	**懐かしい**
I.	Spirituality	Spiritual feeling	Sentiment spirituel	精神感觉	Sentimento espiritual	**スピリチュアルな**
