# Supplementary material for: What emotions are elicited by smells in Japanese people? Emotional measurement using a universal scale in Japanese
Source: PLoS One. 2025 May 13;20(5):e0323206. doi: 10.1371/journal.pone.0323206 (PMC12074331; doi:10.1371/journal.pone.0323206)
Supplement: S1 Appendix — (PDF) [file pone.0323206.s001.pdf]

必須 SC7.
最近、日常生活ではどのくらいにおいを感じますか？
以下の項目についてあてはまるものをそれぞれお知らせください。（それぞれ１つずつ選択）

How much odor do you smell in your daily life these days? Please let us know each of the following items that apply to you. (Select one for each)

ヨコに回答→		わかる I know it.	時々わかる Sometimes I know it.	わからない I don't know it.	最近嗅いでいない 嗅いだことがない I have not smelled or have not smelled recently it.
炊けたご飯	Cooked rice	○	○	○	○
味噌	Miso	○	○	○	○
海苔	Nori (seaweed)	○	○	○	○
醤油	Soy sauce	○	○	○	○
パン屋	Bakery	○	○	○	○
バター	Butter	○	○	○	○
カレー	Curry	○	○	○	○
炒めたニンニク	Sauteed garlic	○	○	○	○
みかん	Japanese mandarin	○	○	○	○
イチゴ	Strawberry	○	○	○	○
ヨコに回答→		わかる	時々わかる	わからない	最近嗅いでいない 嗅いだことがない
緑茶	Green tea	○	○	○	○
コーヒー	Coffee	○	○	○	○
チョコレート	Chocolate	○	○	○	○
家庭用ガス	Household gas	○	○	○	○
生ごみ	Food waste	○	○	○	○
材木	Lumber	○	○	○	○
汗	Sweat	○	○	○	○
糞便	Feces	○	○	○	○
花	Flower	○	○	○	○
香水	Perfume	○	○	○	○

Note: The scores for the choices were as follows: "I know it.": 2 points; "Sometimes I know it.": 1 point; "I don't know it.": 0 point. "I have not smelled or have not smelled recently it." was also zero, and the full score for the participant who chose this option was two points less per option. In other words, the full score varied from participant to participant (in addition, participants who selected more than 10 of this option, "I have not smelled or have not smelled recently it," were excluded). Based on this criterion, the score relative to the full score was indicated by %.
