# Supplementary material for: What emotions are elicited by smells in Japanese people? Emotional measurement using a universal scale in Japanese
Source: PLoS One. 2025 May 13;20(5):e0323206. doi: 10.1371/journal.pone.0323206 (PMC12074331; doi:10.1371/journal.pone.0323206)
Supplement: S2 Appendix — (PDF) [file pone.0323206.s002.pdf]

必須 SC8.

あなたの嗅ぎ取れる匂いの程度は、以下のスケールで例えるとどのくらいですか？

※印象でお答えください。

How would you rate the degree of odor you can smell on the following scale? Please give your impression.

※カーソルを左右に動かしてお答えください。 Please move your cursor to answer.

全く
わからない

十分わかる

I do not recognize it at all.

I recognize it fully.
