# Supplementary material for: What emotions are elicited by smells in Japanese people? Emotional measurement using a universal scale in Japanese
Source: PLoS One. 2025 May 13;20(5):e0323206. doi: 10.1371/journal.pone.0323206 (PMC12074331; doi:10.1371/journal.pone.0323206)
Supplement: S1 Table — (PDF) [file pone.0323206.s003.pdf]

S1 Table. Factor-correlation matrix.

	ML1	ML2	ML3	ML4	ML5	ML6	ML7
ML1	1.00						
ML2	-0.40	1.00					
ML3	0.66	-0.03	1.00				
ML4	0.50	0.17	0.69	1.00			
ML5	0.59	0.04	0.69	0.54	1.00		
ML6	0.56	0.19	0.57	0.67	0.61	1.00	
ML7	0.25	0.25	0.26	0.30	0.23	0.54	1.00

ML1–7 correspond to the extracted factors shown in Table 5.
