# Supplementary material for: What emotions are elicited by smells in Japanese people? Emotional measurement using a universal scale in Japanese
Source: PLoS One. 2025 May 13;20(5):e0323206. doi: 10.1371/journal.pone.0323206 (PMC12074331; doi:10.1371/journal.pone.0323206)
Supplement: S2 Table — (PDF) [file pone.0323206.s004.pdf]

S2 Table. Cross-cultural comparison of extracted factors (colored version of Table 6).

	Europe		North and South America			Asia		
	Geneva, CH	Liverpool, UK	Fayetteville, AR, USA	Davis, CA, USA	Campinas, BR	Beijing, CN	Singapore, SG	**Tokyo, JP**
1	Disgust/ Irritation	Disgust/ Irritation	Disgust/ Irritation	Disgust/ Irritation	Disgust/ Irritation	Disgust/ Irritation	Disgust/ Irritation	**Happiness/Soothing/Attraction**
2	Happiness/ Well-being	Happiness/ Well-being	Happiness/ Well-being	Happiness/ Well-being	Happiness/ Well-being	Happiness/ Well-being	Happiness/ Well-being	**Unpleasant / Anxiety**
3	Sensuality/ Desire	Sensuality/ Desire	Sensuality/ Desire	Sensuality/ Desire	Sensuality/ Desire	Sensuality/ Desire	Sensuality/ Desire	**Vitality**
4	Energy	Energy	Energy	Energy	Energy	Energy	Energy	**Hunger / Thirst**
5	Soothing/ Peacefulness	Soothing/ Peacefulness	Soothing/ Peacefulness	Soothing/ Peacefulness	Soothing/ Peacefulness	Soothing/ Peacefulness	Negative feelings	**Sensuality / Desire**
6	Sensory pleasure	Hunger/ Thirst	Hunger/ Thirst	Hunger/ Thirst	Hunger/ Thirst	Arousal	Intellectual stimulation	**Nostalgia**
7		Nostalgia		Negative feelings	Nostalgia	Melancholy	**Sprituality**	**Sprituality**

Factors with similar or related characteristics are presented in the same color font. Reprinted from a previous study on the UniGEOS [1], except for Japan.

Reference

1. Ferdenzi C, Delplanque S, Barbosa P, Court K, Guinard JX, Guo T, et al. Affective semantic space of scents. Towards a universal scale to measure self-reported odor-related feelings. Food Qual Prefer. 2013;30(2):128-38.
